# Supplementary material for: A curated transcriptome dataset collection to investigate inborn errors of immunity
Source: F1000Res. 2019 Aug 30;8:188. Originally published 2019 Feb 15. [Version 2] doi: 10.12688/f1000research.18048.2 (PMC6749933; doi:10.12688/f1000research.18048.2)
Supplement: Peer review history

# Peer review: Reviewer response for version 2

**DOI:** 10.5256/f1000research.22465.r53217

**Published:** 2019-09-17

**Authors:** John B. Ziegler

**Affiliations:** 1 School of Women's & Children's Health, University of New South Wales, Sydney, NSW, Australia; 2 Department of Immunology & infectious Diseases, Sydney Children's Hospital, Sydney, Australia

All is now in order with significant improvements in response to peer review.

I confirm that I have read this submission and believe that I have an appropriate level of expertise to confirm that it is of an acceptable scientific standard.

# Peer review: Reviewer response for version 2

**DOI:** 10.5256/f1000research.22465.r53218

**Published:** 2019-09-09

**Authors:** Bertrand De Meulder

**Affiliations:** 1 European Institute for Systems Biology & Medicine, Université de Lyon, Lyon, France; 2 Association EISBM, Vourles, France

I feel my comments have been taken into account and hence approve the indexing of this manuscript.

I confirm that I have read this submission and believe that I have an appropriate level of expertise to confirm that it is of an acceptable scientific standard.

# Peer review: Reviewer response for version 1

**DOI:** 10.5256/f1000research.19737.r45485

**Published:** 2019-03-28

**Authors:** John B. Ziegler

**Affiliations:** 1 School of Women's & Children's Health, University of New South Wales, Sydney, NSW, Australia; 2 Department of Immunology & infectious Diseases, Sydney Children's Hospital, Sydney, Australia

This is a useful tool to examine publicly available gene transcript data.

 The paper would be more useful if it included more detailed instructions for its use, perhaps including screenshots to illustrate the text.

 The location of the PubMed link of each sample wasn’t immediately obvious to this reader. I now realise it is accessed by clicking a part of the PubMed.gov icon.

 The meaning of the term “ranking” becomes apparent with use but it would not be burdensome to more experienced readers to have it defined or explained.

 The acronym for induced pluripotent stem cells (iPS) should be defined with first use.

 The sample set “Whole Blood Transcriptional Modules generated on Illumina Hu-6 V2 Beadchips. GSE29536” is listed with the disease category “Immunodeficiencies” but the data presented appears to include none from immunodeficiency settings. I see transcripts from infections, SLE, diabetes, Still’s disease. I can’t get more detail of the study because the PubMed link is wrong. The link provided is https://www.ncbi.nlm.nih.gov/pubmed/24069364 which is actually the link for other listed samples ( GSE51404 and GSE51405). I haven’t verified all links in the table.

 If improvements such as those above were addressed I believe the paper would be very useful to those conducting immunological research.

 If improvements such as those above were addressed I believe the paper would be very useful to those conducting immunological research.

I confirm that I have read this submission and believe that I have an appropriate level of expertise to confirm that it is of an acceptable scientific standard, however I have significant reservations, as outlined above.

# Response

**Published:** 2019-08-27

**Authors:** Salim Bougarn

**Affiliations:** Sidra Medicine, Qatar

Dear Dr. Ziegler,

We are  thankful to Dr. Ziegler  for his positive feed back regarding our manuscript and for the careful revision. We respond to the specific comments and describe the introduced changes to the new version below in the text and in bold.  

The paper would be more useful if it included more detailed instructions for its use, perhaps including screenshots to illustrate the text.

**We have updated a link to the tutorial (http://pid.gxbsidra.org/dm3/tutorials.gsp) as well as a reference to a paper describing [Speake et. al) on how to use GXB with detailed instructions including screenshots.**

The location of the PubMed link of each sample wasn’t immediately obvious to this reader. I now realise it is accessed by clicking a part of the PubMed.gov icon.

The meaning of the term “ranking” becomes apparent with use but it would not be burdensome to more experienced readers to have it defined or explained.

**The ranking was explained in material and methods section in the paper**

The acronym for induced pluripotent stem cells (iPS) should be defined with first use.

**We have added to the paper the definition of iPS.**

The sample set “Whole Blood Transcriptional Modules generated on Illumina Hu-6 V2 Beadchips. GSE29536” is listed with the disease category “Immunodeficiencies” but the data presented appears to include none from immunodeficiency settings. I see transcripts from infections, SLE, diabetes, Still’s disease. I can’t get more detail of the study because the PubMed link is wrong. The link provided is https://www.ncbi.nlm.nih.gov/pubmed/24069364 which is actually the link for other listed samples ( GSE51404 and GSE51405). I haven’t verified all links in the table.

**We have removed the GSE29536 from the instance and the datanote and modified the total number of datasets retrieved and loaded into GXB. But also, we modified the table 1 and figure 1.**

**We have checked and verified all PubMed links in other datasets.**

If improvements such as those above were addressed I believe the paper would be very useful to those conducting immunological research.

**The requested improvements have been incorporated in the paper.**

**Please note that other minor modification updates were done on the manuscript, such as the total number of datasets found in the GEO database using the criteria mentioned in the paper.**

# Peer review: Reviewer response for version 1

**DOI:** 10.5256/f1000research.19737.r45968

**Published:** 2019-03-21

**Authors:** Bertrand De Meulder

**Affiliations:** 1 European Institute for Systems Biology & Medicine, Université de Lyon, Lyon, France; 2 Association EISBM, Vourles, France

This article presents a tool to gather and make preliminary analyses on a set of curated sequencing datasets related to primary immunodeficiencies. 

 Regarding replication, I could not reproduce the list of datasets with the criteria that are mentioned in the text. I would have to believe that the authors are indeed reporting their results, and that the manual curation of the dataset list was accurate.  

 The tool that is presented has a number of issues:

- The list of datasets is rather small, and the authors do not mention whether they plan on adding datasets to the list,
- The tutorials on how to use the tools are only available to registered users, and I could not find how to register
- Graphical issues are present when displaying the charts, both in-tool and in png images.

I confirm that I have read this submission and believe that I have an appropriate level of expertise to confirm that it is of an acceptable scientific standard, however I have significant reservations, as outlined above.

# Response

**Published:** 2019-08-27

**Authors:** Salim Bougarn

**Affiliations:** Sidra Medicine, Qatar

Dear Bertrand, 

 We are  thankful to Dr. De Meulder for his positive feed back regarding our manuscript and for the careful revision. We respond to the specific comments and describe the introduced changes to the new version below in the text and in bold.  

 Regarding replication, I could not reproduce the list of datasets with the criteria that are mentioned in the text. I would have to believe that the authors are indeed reporting their results, and that the manual curation of the dataset list was accurate.  

**This is not related to GXB but on the pubmed search criteria. Also, please note that the GEO database is growing over time and for your attention the query was run in date 2017-2-1. I have updated in the paper the total number of datasets found in the GEO database using the criteria mentioned in the paper.**

 The tool that is presented has a number of issues:

- The list of datasets is rather small, and the authors do not mention whether they plan on adding datasets to the list,

**The curation of the datasets requires substantial effort so we decide to freeze the addition of dataset for this version. We are planning to update the datasets in a future version of the paper. A sentence was added to the material and method in the paper.**

- The tutorials on how to use the tools are only available to registered users, and I could not find how to register

**The tutorial is available also for non registered user via the following link: http://pid.gxbsidra.org/dm3/tutorials.gsp. The link in the paper was modified. **

- Graphical issues are present when displaying the charts, both in-tool and in png images.

**It is not clear what graphical issue exactly you encounter. It is possible to hide info panel information to resize the graphs and get a better visualization.**

****

**Please note that the GSE29536 datasets was removed from the instance and the datanote (Table 1). The figure 1 was slightly modified.  Also, the total number of datasets retrieved, selected and loaded to GXB was modified.**
